# Early parasite clearance following artemisinin-based combination therapy among Ugandan children with uncomplicated *Plasmodium falciparum* malaria

**DOI:** 10.1186/1475-2875-13-32

**Published:** 2014-01-28

**Authors:** Mary K Muhindo, Abel Kakuru, Prasanna Jagannathan, Ambrose Talisuna, Emmanuel Osilo, Francis Orukan, Emmanuel Arinaitwe, Jordan W Tappero, Frank Kaharuza, Moses R Kamya, Grant Dorsey

**Affiliations:** 1 Infectious Diseases Research Collaboration, Mulago Hospital Campus, PO Box 7475, Kampala, Uganda; 2 Department of Medicine, San Francisco General Hospital, University of California, San Francisco, CA, USA; 3 University of Oxford/ KEMRI/ Wellcome Trust Research Programme, Nairobi, Kenya; 4 Global AIDS Program, Centers for Disease Control and Prevention, Atlanta, GA, USA; 5 Centers for Disease Control and Prevention, Kampala, Uganda; 6 Department of Medicine, Makerere University College of Health Sciences, Kampala, Uganda

**Keywords:** Malaria, *Plasmodium falciparum*, Artemisinin-based combination therapy, Parasite clearance, Artemether-lumefantrine, Dihydroartemisin-piperaquine

## Abstract

**Background:**

Artemisinin-based combination therapy (ACT) is widely recommended as first-line therapy for uncomplicated *Plasmodium falciparum* malaria worldwide. Artemisinin resistance has now been reported in Southeast Asia with a clinical phenotype manifested by slow parasite clearance. Although there are no reliable reports of artemisinin resistance in Africa, there is a need to better understand the dynamics of parasite clearance in African children treated with ACT in order to better detect the emergence of artemisinin resistance.

**Methods:**

Data from a cohort of Ugandan children four to five years old, enrolled in a longitudinal, randomized, clinical trial comparing two leading ACT, artemether-lumefantrine (AL) and dihydroartemisinin-piperaquine (DP), were analysed. For all episodes of uncomplicated *P. falciparum* malaria over a 14-month period, daily blood smears were performed for three days following the initiation of therapy. Associations between pre-treatment variables of interest and persistent parasitaemia were estimated using multivariate, generalized, estimating equations with adjustment for repeated measures in the same patient.

**Results:**

A total of 202 children were included, resulting in 416 episodes of malaria treated with AL and 354 episodes treated with DP. The prevalence of parasitaemia on days 1, 2, and 3 following initiation of therapy was 67.6, 5.6 and 0% in those treated with AL, and 52.2, 5.7 and 0.3% in those treated with DP. Independent risk factors for persistent parasitaemia on day 1 included treatment with AL *vs* DP (RR = 1.34, 95% CI 1.20-1.50, p < 0.001), having a temperature ≥38.0°C *vs* < 37.0°C (RR = 1.19, 95% CI 1.05-1.35, p = 0.007) and having a parasite density >20,000/μL *vs* <4,000/μL (RR = 3.37, 95% CI 2.44-4.49, p < 0.001). Independent risk factors for having persistent parasitaemia on day 2 included elevated temperature, higher parasite density, and being HIV infected.

**Conclusions:**

Among Ugandan children, parasite clearance following treatment with AL or DP was excellent with only one of 752 patients tested having a positive blood slide three days after initiation of therapy. The type of ACT given, pre-treatment temperature, pre-treatment parasite density and HIV status were associated with differences in persistent parasitaemia, one or two days following therapy.

**Trial registration:**

Current Controlled Trials Identifier NCT00527800.

## Background

Artemisinin-based combination therapy (ACT) is currently recommended for the treatment of uncomplicated *Plasmodium falciparum* malaria by the World Health Organization and has been adopted as first-line therapy in most malaria-endemic countries [1]. Marked reductions in malaria-associated morbidity and mortality in many parts of the world have been attributed to the replacement of failing monotherapies with highly effective ACT and the scale-up of vector control measures [2]. However the recent emergence and/or spread of artemisinin resistance in parts of Southeast Asia [3-6] poses a serious threat to malaria control efforts. Although artemisinin resistance has not yet been reported in Africa, there is a historical precedence of resistance to older monotherapies emerging in Asia and spreading to Africa with devastating effects [7-9]. Thus early and accurate detection of artemisinin resistance in African populations will be critical for the implementation of containment efforts.

The phenotype of artemisinin resistance is characterized by a significant delay in parasite clearance following initiation of therapy [3-6]. As there are no reliable molecular markers of resistance and *in vitro* correlates have been inconsistent [10], surveillance of artemisinin resistance to date has relied on *in vivo* studies to measure early clearance of peripheral parasitaemia by microscopy. The use of sampling multiple times a day at measured time points to estimate the rate of parasite clearance has been proposed as an accurate and reliable method for the early detection of artemisinin resistance [11]. However, this approach may be difficult to implement in settings of routine *in vivo* drug efficacy studies among outpatients. An alternative approach is to measure the proportion of patients with detectable parasitaemia one, two or three days after the initiation of therapy [12,13]. Although this alternative approach is simple to implement, there are multiple factors that could be associated with parasite clearance independent of artemisinin resistance, which may confound the interpretation of results.

In this study the proportion of patients with detectable parasitaemia one, two and three days after initiation of therapy were measured in a cohort of Ugandan children randomized to two different ACT regimens. The objective of the study was to identify factors associated with early parasite clearance in a setting where artemisinin resistance has not yet occurred. This information will be important for the interpretation of future surveillance studies aimed at identifying early signs of artemisinin resistance in African populations.

## Methods

### Study design, site and population

This study was part of a larger open-label, randomized trial conducted at Tororo, an area in Eastern Uganda with high malaria transmission intensity [14]. The methods of the main study have been described previously [15,16]. Briefly, convenience sampling was used to enrol a cohort of HIV infected and uninfected children referred to a dedicated study clinic from an adjacent post-natal clinic at Tororo District Hospital. Eligibility criteria included: 1) age six weeks to 12 months, 2) documented HIV status of mother and child, 3) agreement to come to the study clinic for any febrile episode or other illness, 4) residence within a 30-km radius of the study clinic, 5) absence of active medical problem requiring inpatient evaluation at the time of screening, and, 6) provision of informed consent. At enrolment, all study participants received a long-lasting, insecticide-treated bed net (ITN).

### Follow-up of study participants

Subjects were followed for all of their medical problems at a dedicated study clinic open seven days a week and parents/guardians were encouraged to bring their children to the study clinic whenever they were ill. HIV-infected children were prescribed daily trimethoprim-sulphamethoxazole (TS) prophylaxis. Medications with anti-malarial activity were avoided for the treatment of non-malarial illnesses. Study participants were followed until they reached five years of age or met one of the following criteria for early study withdrawal: 1) movement out of the study area, 2) inability to be located for >60 consecutive days, 3) withdrawal of informed consent, 4) inability to adhere to the study schedule and procedures, or, 5) inability to tolerate the drugs used for malaria treatment.

### Malaria diagnosis and treatment

Children who presented to the study clinic with a documented fever (tympanic temperature ≥38.0˚C) or history of fever in the previous 24 hours had blood obtained by finger prick for a thick smear. If the thick smear was positive, the patient was diagnosed with malaria regardless of parasite density. Children who were aged ≥ four months and weighing ≥5 kg were randomized to receive either artemether-lumefantrine (AL) or dihydroartemisinin-piperaquine (DP) at the time they got their first episode of uncomplicated malaria. Study participants received the same treatment regimen for all subsequent episodes of uncomplicated malaria. Study drugs were given according to weight-based guidelines for fractions of tablets as follows: AL (tablets of 20 mg of artemether and 120 mg of lumefantrine; Coartem; Novartis), administered as one (5–14 kg) or two (15–24 kg) tablets given twice daily for three days; and DP (tablets of 40 mg of dihydroartemisinin and 320 mg of piperaquine; Duocotecxin; Holley Pharm), targeting a total dose of 6.4 and 51.2 mg/kg of dihydroartemisinin and piperaquine, respectively, given in three equally divided daily doses to the nearest one-quarter tablet. Study drugs were crushed, mixed with water and administered to the patient. Patients were given a glass of milk after each dose. The first daily dose of study drugs was directly observed at the study clinic. After each dose, children were observed for 30 minutes, and the dose was re-administered if vomiting occurred. For children treated with AL, the second daily dose was packaged and the parent or guardian was given verbal instructions for proper administration of the medication at home with clear emphasis on when the evening dose should be given. Episodes of complicated malaria and treatment failures occurring within 14 days of initiating treatment were treated with quinine. All children with malaria were followed up on days 1, 2, 3, 7, 14, 21, and 28 following enrolment.

### Laboratory methods

Thick and thin blood smears were stained with 2% Giemsa for 30 minutes and read by trained laboratory technologists who were not involved in direct patient care. Parasite densities were calculated from thick blood smears by counting the number of asexual parasites per 200 leukocytes (or per 500 leukocytes, if the count was <10 asexual parasites/200 leukocytes), assuming a leukocyte count of 8,000/μl. A blood smear was considered negative when the examination of 100 high power fields did not reveal asexual parasites. For quality control, all slides were read by a second reader. An independent third reader settled any discrepancies between the first and second readings. Laboratory technicians were blinded to the study participants’ treatment assignments. Thin smears were used to determine the parasite species.

### Statistical methods

Data were double entered into an Access database and analysed using Stata version 11 (Stata Corp, College Station, TX, USA). This study included only data from episodes of uncomplicated *P. falciparum* malaria diagnosed between October 2011 and December 2012 when routine blood smears were added on day 1 of malaria follow-up. During this period, all children remaining in the cohort study were between 47 and 60 months of age. The primary outcome of interest was the proportion of patients who remained parasitaemic by microscopy one, two and three days following initiation of therapy. Secondary outcomes included persistence of fever (either subjective fever in the previous 24 hours or tympanic temperature ≥38.0°C,) during the first three days following initiation of therapy and 28-day standardized WHO treatment outcomes unadjusted by genotyping. The treatment outcomes were classified as: adequate clinical and parasitological response (all blood smears negative after day 3); early treatment failure (presence of danger signs or complicated malaria with a positive blood smear within three days of treatment, or day 2 parasite density > day 0 parasite density, or day 3 parasite density >25% of day 0 parasite density, or positive blood smear on day 3 with a tympanic temperature ≥38.0°C); late clinical failure (first positive blood smear after day 3 in the presence of fever); and late parasitological failure (first positive blood smear after day 3 in the absence of fever). Risk factors of interest included the ACT regimen (AL *vs* DP); mg/kg dosing of anti-malarials; treatment episode number; pre-treatment temperature, parasite density and haemoglobin; age; gender; and HIV status. We explored the relationship between pre-treatment parasite density and proportion with parasitaemia and it was nonlinear. We thus created a categorical variable for parasite density to create the most parsimonious model that best fit the distribution of the data. Associations between risk factors of interest and early persistent parasitaemia were estimated using generalized estimating equations with robust standard errors and adjustment for repeated measures in the same patient. Risk factors associated with the outcome of interest with a p-value of < 0.05 in univariate analyses were included in the final multivariate model, with the exception of age and gender which were also included.

### Ethical approval

Informed consent was obtained from the parents or legal guardian of all study participants. The study protocol was approved by the Uganda National Council of Science and Technology and the institutional review boards of the University of California, San Francisco, Makerere University, the University of Washington, and the Centers for Disease Control and Prevention.

## Results

### Study profile and characteristics of the malaria episodes

**Figure 1:**
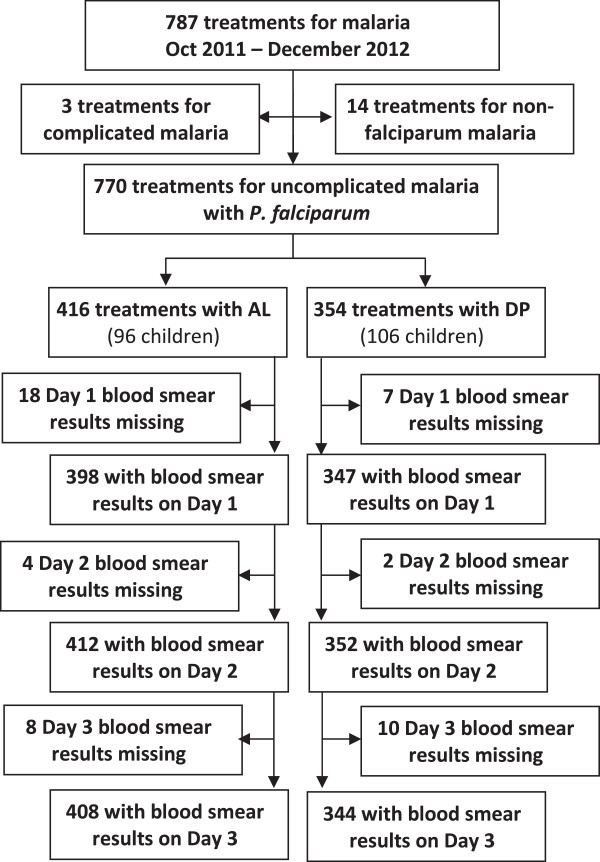
**Trial profile.** AL = artemether-lumefantrine, DP = dihydroartemisinin-piperaquine.

A total of 787 episodes of malaria were treated in 202 study participants during the study period of October 2011 to December 2012. Three episodes of complicated malaria treated with quinine and 14 episodes of malaria caused by non-falciparum species were excluded from the analysis (Figure 1). Of 770 uncomplicated malaria episodes due to *P. falciparum*, 416 episodes were treated with AL and 354 episodes were treated with DP. The mean age, temperature, and haemoglobin level was similar in both treatment arms (Table 1). Episodes treated with DP had a higher parasite density compared to those treated with AL (20,295 *vs* 13,258 parasites/μl, p = 0.004). A total of 38 (4.9%) episodes occurred in HIV-infected children and were equally distributed between the two treatment arms.

**Table 1:** Baseline characteristics of all episodes of uncomplicated falciparum malaria

**Characteristic**	**Treatment arm**	
	**AL (n = 416)**	**DP (n = 354)**
Patient age in months, mean (range)	56 (48–60)	56 (47–60)
Gender, number female (%)	210 (50.5)	141 (39.8)
Dosing of artemisinin drug in mg/kg, median (range)	14.3 (8.3-16.6)	7.2 (5.8-8.5)
Dosing of partner drug in mg/kg, median (range)	85.7 (50.0-99.3)	57.5 (46.2-67.9)
Temperature °C, mean (SD)	37.6 (1.0)	37.7 (1.0)
Parasite density per μL, geometric mean	13,258	20,295
Haemoglobin g/dL, mean (SD)	11.1 (1.5)	11.2 (1.5)
HIV infected, n (%)	18 (4.3%)	20 (5.7%)
Treatments per child, median (range)	3 (1–13)	2 (1–13)

### Malaria treatment outcomes

On day one following initiation of anti-malarial treatment, a total of 450 of 745 (60.4%) episodes with blood smears done had persistent asexual parasitaemia by microscopy and the prevalence was higher in those treated with AL compared to DP (67.6 *vs* 52.2%, *p* < 0.001). On day two following initiation of anti-malarial treatment, a total of 43 of 764 (5.6%) episodes with blood smears done had persistent asexual parasitaemia by microscopy and the prevalence was similar between the two ACT treatment arms. On day three following initiation of anti-malarial treatment, only one of 752 (0.1%) episodes with blood smears done had persistent parasitaemia (Table 2). This patient was treated with DP and had a parasite density of only 16 asexual parasites/μL on day three which cleared by day seven when the next blood smear was done.

**Table 2:** Response to anti-malarial therapy

**Characteristic**	**Treatment arm**		**p-value**
	**AL (n = 416)**	**DP (n = 354)**	
**Parasite persistence, n (%)**			
Positive blood smear day 1	269 (67.6%)	181 (52.2%)	<0.001
Positive blood smear day 2	23 (5.6%)	20 (5.7%)	0.92
Positive blood smear day 3	0	1 (0.3%)	N/A
**Fever persistence, n (%)**			
Febrile* day 1	124 (30.0%)	65 (18.4%)	0.002
Febrile* day 2	8 (1.9%)	11 (3.1%)	0.28
Febrile* day 3	7 (1.7%)	7 (2.0%)	0.78
**28-day WHO treatment outcome, n (%)**			
Lost to follow-up	16 (3.9%)	13 (3.7%)	
Early treatment failure	0	1 (0.3%)	
Late parasitological failure	137 (32.9%)	22 (6.2%)	< 0.001
Late clinical failure	74 (17.8%)	7 (2.0%)	
Adequate clinical and parasitological response	189 (45.4%)	311 (87.9%)	

*Subjective fever in the previous 24 hours or temperature ≥38.0°C (tympanic).

Patients treated with AL were more likely to be febrile on day 1 following initiation of anti-malarial treatment compared to those treated with DP (30 *vs* 18.4%, respectively, *P* = 0.002). There was no significant difference between the two ACT treatment arms in the prevalence of fever on day two and three following initiation of anti-malarial therapy (Table 2). Considering 28-day WHO treatment outcomes, there was only one early treatment failure, which occurred in a patient treated with DP due to an increase in the parasite density from 12,720 asexual parasites/μL on day 0 to 18,960 asexual parasites/μL on day two. Interestingly, when this patient returned to clinic on day three the blood smear was negative and therefore rescue therapy was not given. Patients treated with DP were significantly more likely to have an adequate clinical and parasitological response compared to those treated with AL (87.9 *vs* 45.4%, p < 0.001) (Table 2).

### Risk factors associated with a persistent parasitaemia on days one and two following initiation of anti-malarial therapy

In univariate analyses, the anti-malarial treatment regimen administered, pre-treatment temperature, and pre-treatment parasite density were significantly associated with persistent parasitaemia on day one following initiation of anti-malarial therapy. In the final multivariate model the following factors were significantly associated with an increased risk of persistent parasitaemia on day one: treatment with AL compared to DP (RR = 1.34, 95% CI 1.20-1.50, p < 0.001); pre-treatment temperature ≥38.0 compared to <37.0 (RR = 1.19, 95% CI 1.05-1.35, p = 0.007); and pre-treatment parasite density >20,000/μL (RR = 3.31, 95% CI 2.44-4.49, p < 0.001) and 4,000-20,000/μL (RR = 2.61, 95% CI 1.89-3.59, p < 0.001) compared to <4,000/μL (Table 3). In univariate analyses pre-treatment temperature, pre-treatment parasite density, and HIV status were significantly associated with persistent parasitaemia on day two following initiation of anti-malarial therapy. In the final multivariate model the following factors were significantly associated with an increased risk of persistent parasitaemia on day two: pre-treatment temperature ≥37.0 (RR = 2.32, 95% CI 1.00-5.38, p = 0.049); pre-treatment parasite density >20,000/μL (RR = 5.09, 95% CI 2.19-11.9, p < 0.001); and being HIV infected (RR = 3.93, 95% CI 1.74-8.88, p = 0.001) (Table 4).

**Table 3:** Associations between variables of interest and a positive blood slide on day 1

**Variable**	**Categories**	**Risk of positive blood smear on day 1**	**Univariate analysis**		**Multivariate analysis**	
			RR* (95% CI)	**p-value**	**RR**^**† **^**(95% CI)**	**p-value**
**Anti-malarial treatment**	DP	52.2%	1.0 (reference)	-	1.0 (reference)	-
	AL	67.6%	1.30 (1.13-1.49)	<0.001	1.35 (1.21-1.50)	<0.001
**mg/kg dosing of anti-malarial**	≥ median (DP)	55.0%	1.0 (reference)	-	Not included in final model	
	< median (DP)	49.4%	0.96 (0.78-1.18)	0.68		
	≥ median (AL)	64.3%	1.0 (reference)	-		
	< median (AL)	70.7%	1.11 (0.96-1.28)	0.16		
**Temperature °C**	< 37.0	48.2%	1.0 (reference)	-	1.0 (reference)	-
	37.0-37.9	59.7%	1.22 (1.03-1.44)	0.02	1.09 (0.96-1.24)	0.19
	≥ 38.0	71.2%	1.47 (1.25-1.73)	<0.001	1.17 (1.04-1.33)	0.01
**Parasite density per μL**	< 4,000	21.3%	1.0 (reference)	-	1.0 (reference)	-
	4,000-20,000	57.4%	2.64 (1.93-3.63)	<0.001	2.62 (1.90-3.62)	<0.001
	> 20,000	73.5%	3.39 (2.51-4.59)	<0.001	3.31 (2.44-4.49)	<0.001
**Haemoglobin g/dL**	≥ 10	60.0%	1.0 (reference)	-	Not included in final model	
	< 10	64.0%	1.08 (0.92-1.26)	0.33		
**HIV status**	Negative	60.5%	1.0 (reference)	-	Not included in final model	
	Positive	58.3%	0.97 (0.72-1.31)	0.84		
**Episode number**	1-2	59.9%	1.0 (reference)		Not included in final model	
	3-4	57.1%	0.94 (0.82-1.09)	0.42		
	≥ 5	65.0%	1.02 (0.90-1.17)	0.74		

*Relative risk using generalized estimating equations with adjustment for repeated measures in the same patient.

^**†**^Relative risk using generalised estimating equations with adjustment for repeated measures in the same patient, age and gender.

**Table 4:** Associations between variables of interest and a positive blood slide on day 2

**Variable**	**Categories**	**Risk of positive blood smear on day 2**	**Univariate analysis**		**Multivariate analysis**	
			RR* (95% CI)	**p-value**	**RR**^**† **^**(95% CI)**	**p-value**
**Anti-malarial treatment**	DP	5.7%	1.0 (reference)	-	Not included in final model	
	AL	5.6%	1.03 (0.55-1.95)	0.92		
**mg/kg dosing of anti-malarial**	≥ median (DP)	5.8%	1.0 (reference)	-	Not included in final model	
	< median (DP)	5.6%	1.00 (0.41-2.43)	0.99		
	≥ median (AL)	4.0%	1.0 (reference)	-		
	< median (AL)	7.1%	1.74 (0.70-4.38)	0.23		
**Temperature °C**	< 37.0	2.6%	1.0 (reference)	-	1.0 (reference)	-
	37.0-37.9	5.9%	2.35 (0.98-5.59)	0.05	2.32 (1.01-5.31)	0.048
	≥ 38.0	7.8%	3.09 (1.28-7.46)	0.01		
**Parasite density per μL**	< 4,000	1.4%	1.0 (reference)	-	1.0 (reference)	-
	4,000-20,000	1.3%				
	> 20,000	8.3%	5.79 (2.35-14.3)	<0.001	5.12 (2.12-12.37)	<0.001
**Haemoglobin g/dL**	≥ 10	5.4%	1.0 (reference)	-	Not included in final model	
	< 10	6.0%	1.06 (0.46-2.45)	0.89		
**HIV status**	Negative	5.0%	1.0 (reference)	-	1.0 (reference)	-
	Positive	19.4%	3.87 (1.85-8.12)	<0.001	3.69 (1.54-8.84)	0.003
**Episode number**	1-2	6.9%	1.0 (reference)	-	Not included in final model	
	3-4	5.1%	0.74 (0.37-1.48)	0.39		
	≥5	3.7%	0.50 (0.20-1.29)	0.15		

*Relative risk using generalized estimating equations with adjustment for repeated measures in the same patient.

^**†**^Relative risk using generalized estimating equations with adjustment for repeated measures in the same patient, age and gender.

## Discussion

In this study of 770 episodes of uncomplicated falciparum malaria in Ugandan children randomized to therapy with AL or DP, parasite clearance was excellent with only one episode of parasitaemia documented by microscopy three days after initiation of therapy. To better understand the dynamics of early parasite clearance following ACT treatment in an African setting without evidence of artemisinin resistance, several risk factors associated with persistent parasitaemia one and two days after initiation of therapy were identified. Considering persistent parasitaemia on day one, treatment with AL compared to DP, an elevated pre-treatment temperature, and higher pre-treatment parasite density, were independently associated with a significantly increased risk of persistent parasitaemia. Considering persistent parasitaemia on day two, an elevated pre-treatment temperature, higher pre-treatment parasite density and being HIV infected were independently associated with a significantly increased risk of persistent parasitaemia.

The rationale behind ACT is based on the highly potent, but short-acting artemisinin component causing rapid reduction in peripheral parasitaemia followed by the longer-acting partner drug eliminating any remaining parasites [17]. ACT has now become one of the most effective tools for malaria control and the emergence of resistance to the artemisinin class of anti-malarial drugs would pose a major global health problem. Artemisinin resistance was first reported in Western Cambodia and has now emerged or spread to other areas of Southeast Asia [3-6]. Currently the only way to identify artemisinin resistance is through the detection of phenotypic trait of delayed parasite clearance *in vivo* following the initiation of therapy. Thus, there is great interest in surveillance studies of early parasite clearance to provide an "early warning system" for the emergence/spread of artemisinin resistance in areas where this has not be previously documented. However, accurate characterization of the clinical phenotype of delayed parasite clearance may be complicated by a number of factors acting independent of artemisinin resistance, such as drug concentrations, pharmacodynamics properties of the partner drug, pre-treatment parasite density, and host immunity.

In this study, a number of factors were independently associated with persistent parasitaemia on days one and two following the initiation of ACT. A higher pre-treatment parasite density had the strongest association with persistent parasitaemia on days one and two. This finding is expected given that time to parasite clearance is a function of the baseline parasite density [18]. Thus any assessment of early parasite clearance should account for the pre-treatment parasite density. One method proposed to account for pre-treatment parasite density is to estimate the parasite clearance rate, as defined by the slope of the linear portion of the natural logarithm of the parasite clearance curve, rather than simply the proportion of patients who remain parasitaemic following the initiation of therapy [11]. However, this method requires frequent sampling, ideally every six to eight hours or at least every 12 hours until parasite clearance, which may pose considerable challenges in the setting of routine drug efficacy surveillance studies. For clinical studies that continue to rely on less frequently sampling and determine the proportion of patients who remain parasitaemic at different time points, control of pre-treatment parasite density will be essential for monitoring trends in early parasite clearance over time and space.

In this study a higher pre-treatment temperature was also associated with persistent parasitaemia on days one and two. This finding has been reported in another study from Kenya [19] and may be a surrogate marker of a less effective host immune response. In addition, infection with HIV was associated with persistent parasitaemia on day two in this study, providing further evidence of the role of the host immune response on early parasite clearance. HIV infection has been associated with an increased incidence of malaria [20] and increased risk of re-infection following therapy [21]. In a small study from Ethiopian adults, HIV-infected patients had a higher risk of persistent parasitaemia 32 hours after the initiation of artemisinin monotherapy compared to HIV-uninfected patients (six of seven *vs* two of 12, p = 0.003) [22]. Age has also been used as a surrogate marker of anti-malarial immunity and would be expected to influence early parasite clearance. Although the age range for patients enrolled in this study was too narrow for evaluation, other studies have reported associations between younger age and persistent parasitaemia [12,23].

Although early parasite clearance is predominantly a function of artemisinin activity, different formulations of the artemisinin component and partner drugs used in various ACT may also influence early parasite clearance. In this study, treatment with AL was associated with a higher risk of persistent parasitaemia on day one compared to treatment with DP. This finding appears to have some clinical significance as persistent fever on day one was also higher in patients treated with AL compared to DP. Similar findings of a higher risk of persistent parasitaemia on day one in AL compared to DP have also been reported in a study from Kenya [19] as well as a large meta-analysis of studies from Asia and Africa [12].

There were several limitations to this study. As mentioned earlier, blood smear samples were obtained once daily, therefore, it was not possible to accurately estimate the parasite clearance rate as a continuous variable. This study also included patients across a narrow age range (47–60 months) and over a relatively short period of calendar time, thus it was not possible to evaluate age as a potential risk factor or temporal trends in early parasite clearance. Finally, only DP was fully administered as directly observed therapy, as it was given once daily when a child presented to the study clinic. In contrast, AL was given twice daily and only one of the two daily doses was directly observed. If the second dose of AL was not appropriately administered at home, differences in parasite clearance between AL and DP may have been overestimated.

In summary, results from this study provide reassuring evidence that among children living in an area of high malaria endemicity in Uganda, parasite clearance following treatment with AL and DP was excellent. To provide a "baseline" assessment of the dynamics of early parasite clearance in this study population, several factors were associated with persistent parasitaemia at days one and two after the initiation of therapy, including pre-treatment parasite density, pre-treatment temperature, which ACT was given, and HIV status. In order to accurately detect temporal changes in early parasite clearance associated with the emergence of artemisinin resistance in Africa, analyses should control for these risk factors and potentially others when comparing data across studies. Finally, studies with richer parasite density sampling may be required to model the optimum parasite density sampling approaches (daily, six, eight or 12 hourly) to robustly track changes in early parasite clearance in Africa.

## Competing interests

The authors declare that they have no competing interests.

## Authors’ contributions

MKM, AK, MRK, and GD conceived and designed the study. MKM, AK, EO, FO and EA participated in data collection. MKM, AK, MRK, and GD preformed the data analysis. All authors participated in the writing of the manuscript. All authors read and approved the final manuscript.
